# Nasal lysine aspirin challenge in the diagnosis of aspirin - exacerbated respiratory disease

**DOI:** 10.1111/cea.12110

**Published:** 2013-07-29

**Authors:** B Miller, R Mirakian, S Gane, J Larco, A A Sannah, Y Darby, G Scadding

**Affiliations:** 1 Imperial CollegeLondon, UK; 2 RNTNE HospitalLondon, UK

**Keywords:** aspirin, aspirin-exacerbated respiratory disease, asthma, diagnosis, nasal polyps

## Abstract

**Background:**

Aspirin-exacerbated respiratory disease is under-diagnosed and therefore effective and inexpensive therapy with aspirin desensitization is rarely performed.

**Methods:**

We present an audit of 150 patients with difficult to treat nasal polyposis, 132 of whom also had asthma, 131 of whom underwent challenge with the only soluble form of aspirin, lysine aspirin (LAS), to confirm or exclude the diagnosis of aspirin-exacerbated respiratory disease (AERD).

**Results:**

One hundred patients proved positive on nasal challenge, 31 who were negative went onto oral LAS challenge and a further 14 gave positive results, leaving 17 who were negative to a dose equivalent to over 375 mg of aspirin. Nineteen were not challenged because of contraindications.

With the exception of one patient who developed facial angioedema and two patients with > 20% drop in FEV1 (following nasal plus oral challenge) no other severe adverse events occurred. No hospitalization was required for these three patients.

Nasal inspiratory peak flow monitoring was less sensitive to obstruction caused by aspirin than was acoustic rhinometry – which should be employed when aspirin challenge is an outpatient procedure.

**Conclusions:**

Provided patients are carefully chosen and monitored LAS challenge is suitable for ENT day case practice where respiratory physician help with asthma is available and should reduce the under-diagnosis of this condition.

## Introduction

Aspirin-Exacerbated Respiratory Disease (AERD) consists of asthma, nasal and sinus polyps and a respiratory sensitivity to aspirin and non-steroidal anti-inflammatory drugs 1. Patients with this syndrome, often referred to as aspirin triad or Samter’s triad, have progressive inflammatory disease of the upper and lower respiratory tracts 2. The prevalence of aspirin sensitivity ranges from 0.6% to 2.5% of the general population and between 2% and 23% in asthmatic individuals 3,4. The prevalence of aspirin sensitivity in patients with asthma and nasal polyposis has been estimated at 25.6% 5. AERD is therefore not rare in ENT practice since many patients present with initial nasal symptoms, often non-allergic rhinitis,succeeded by nasal polyposis: often severe with a CT scan ‘white out’ and rapidly recurrent following surgery 6. It is however under-diagnosed. The nasal polyp size and the degree of mucosal inflammation are more extensive in aspirin-sensitive than in aspirin-tolerant patients 2. The clinical history alone is not a reliable tool for diagnosing hypersensitivity to aspirin 7 as many asthma patients are advised to avoid aspirin, or the reaction may have occurred many years before and other drugs may have also been taken at the same time. It has been shown that patients with AERD are not solely aspirin-sensitive but their reactivity is to COX-1 inhibitors in general, so reactions occur to many non-steroidal anti-inflammatories 1,8.

There is a need to diagnose aspirin sensitivity: to warn patients accurately about avoidance, to enable the non-sensitive to use therapeutic aspirin and NSAIDS and to employ aspirin desensitization in those who are sensitized.

Unfortunately, there is no suitably sensitive and specific *in vitro* test 9 so drug challenge has to be employed, except where there is a history of recent ingestion without problems (negative) or of adverse reaction to two different NSAID molecules (positive).

Oral or bronchial challenge, starting with a very low dose, usually around 30 mg, and then giving gradually increasing doses can be used 10. Although bronchial challenge can take only 4 h to perform 11, both these methods can result in severe symptoms in over 50% of subjects, especially asthma, and may require hospital admission, emergency treatment, an intravenous line and close monitoring. Since the reaction can be delayed the challenge often requires more than 1 day and thus admission of the subject overnight.

An alternative method, introduced in the 1990s, is that of initial nasal challenge using the only truly soluble form of aspirin, lysine aspirin(L-ASA) 12. This has the advantage that the challenge is directly aimed at possibly aspirin-sensitive polyp tissue. This method is particularly relevant for patients who present to ENT/Rhinology clinics suffering from severe upper airway symptoms resistant to medical treatment and multiple nasal operations. Highly aspirin-sensitive patients should react at low doses with mainly nasal symptoms with very little asthma exacerbation. The less sensitive who tolerate the initial nasal doses are unlikely to be severely affected when they react to subsequent oral challenge. The aim of this study was as follows:

- To explore the feasibility, safety and efficiency of this method if carried out in a day case setting by experienced staff members.
- To establish the first step towards a local nasal aspirin desensitization programme. We report our experience with this method.

## Patients

One hundred and fifty subjects were recruited from our tertiary Rhinology clinic, in which patients with complex respiratory disease are seen. All were symptomatic despite conventional therapy with saline nasal douching, topical corticosteroids and, where effective, leukotriene antagonists. One hundred and twenty had undergone sinus surgery with a mean of 3.8 operations. Two had non-allergic rhinitis, the remainder had chronic rhinosinusitis with nasal polyposis. Asthma was associated in 132. Seven had a definitive history of AERD and underwent challenge preparatory to desensitization, 86 had a suggestive history (reaction to one NSAID only or a doubtful reaction) and 53 had not taken any recent aspirin or NSAID (four had tolerated one NSAID, but not within 6 months).

Intranasal corticosteroids were stopped for 1 week prior to challenge, all other treatments, especially anti-asthma therapy and including montelukast 13 were continued.

### Exclusion criteria

Since polyp expansion and nasal airway monitoring 10,12 is vital to assess the reaction, patients with grade 3 or larger polyps were treated, either medically with oral corticosteroids and betamethasone drops, or surgically, to reduce polyp size prior to the challenge. At least a month was permitted to elapse between polyp reduction and subsequent challenge.

**Figure 1:**
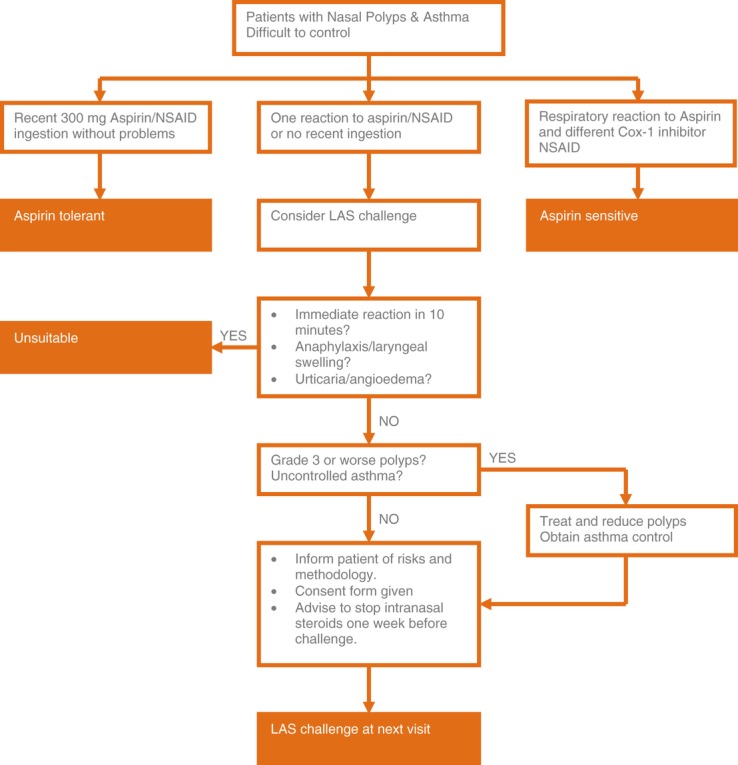
Decision algorithm for consideration of lysine aspirin nasal challenge.

Patients younger than 18 years and older than 65 years of age were excluded. Those with a definite history of anaphylaxis or of urticaria/angioedema to aspirin were also excluded, as were those who had a reaction occurring within a few minutes, as this was likely to be IgE-mediated. Those who recently (last 6 months) tolerated aspirin or a Cox 1 NSAID were regarded as tolerant and were not challenged. Patients with chronic urticaria and patients with unstable cardiovascular conditions or severe or unstable/brittle asthma (FEV1 < 65% on preventative inhalers) were excluded. Patients with uncontrolled asthma were later included if asthma control had been established by guideline – directed treatment. A decision algorithm is given in Fig. 1.

### Control subjects

Six non-rhinitic, non-asthmatic control subjects (3 males, age range 35–65) were tested with 60 mg nasal lysine aspirin as a single dose, following a negative saline challenge.

## Methods

### Ethics

Ethical approval was unnecessary for this audit of our routine diagnostic practice; however, in fact all the subjects received written information at least one week beforehand and signed a consent form which included aspirin challenge to take part in a study of the genetics of aspirin hypersensitivity which will be reported later.

### Subjective evaluation

Each patient assessed symptoms of running, blocking, itching and sneezing, experienced before and during the challenge using a validated 10 cm visual analogue scale.

### Objective evaluation

#### Nasal endoscopy

A rigid nasal endoscope with 0° or 30° angle was gently inserted into each nostril, examining the inferior and middle meatus, nasopharynx, septal anatomy and endonasal mucosal state and noting the grade of polyps in each nostril according to the Lund–Kennedy score 15.

#### Acoustic rhinometry

The nasal airway was monitored using the acoustic rhinometer according to the guidelines for nasal challenge 16. This is a measure of nasal patency, measuring echoes of sound impulses sent into one nostril. The measurement provides information on the nasal luminal anatomical structures, either as a measure of nasal volume over a standard distance into the nostril or as the minimal cross-sectional area within the nasal cavity. The measurement was performed in each nasal cavity separately but the combined score was used to assess changes in patency, both at the minimal cross-sectional area (A min) and also using the total nasal volume from 0 to 12 cm.

### Nasal inspiratory peak flow

This was measured using the Youlten peak flow meter (Clement Clark International), which is attached to an anaesthesia mask which was chosen for each patient so that it was not too large preventing leakage of air, nor too small compressing the nose with impairment of nasal inspiration.

The patient was asked to blow the nose at the beginning of the study then to exhale maximally, after which the mask was placed over the nose and mouth with an airtight seal. The patient was then asked to inspire forcefully through the nose with closed lips. Lip closure was verified during the PNIF test by inspection through the transparent anaesthesia mask.

The nasal flow was expressed in litres per minute, and consecutive measurements were performed taking the best of three outcomes 17.

### Spirometry

Lower respiratory function was evaluated using a spirometer (Model Vitalograph 2160, Maids Moreton, UK), complying with the European Respiratory Society Recommendations. The FVC (% predicted), FEV1 (% predicted) and FVC/FEV1 (%) were recorded. A drop of 20% FEV1 was considered positive.

### Placebo

0.9% saline solution at room temperature from freshly opened 5–10 mL ampoules was used.

### Lysine aspirin

A solution of 900 mg of lysine aspirin (Sanofi, Paris, France) – which is equivalent to 500 mg of acetyl salicylic acid (aspirin) – was freshly prepared at the start of the procedure by dissolving the contents of one sachet (ASPEGIC 500 mg, Sanofi – Aventis, Ditto, France) in 10 mL saline. Lysine aspirin is known to be more water soluble than aspirin (40% vs. 0.3%) and is non-irritant. Fresh supplies were made after 4 h since the solution is unstable.

### Challenge procedure

The patient sat quietly in the laboratory for an initial stabilization period of 15 min then baseline measurements of symptoms, nasal airway and lower airway were taken. An initial single blind challenge with normal saline, as placebo, was carried out administering 100 μL each side via a pipette with the patient in the head upside down position for 1 min, as the drops are delivered directly to the polyps in this way. Delivering the solution via a pipette was found to be more accurate than spray delivery.

**Figure 2:**
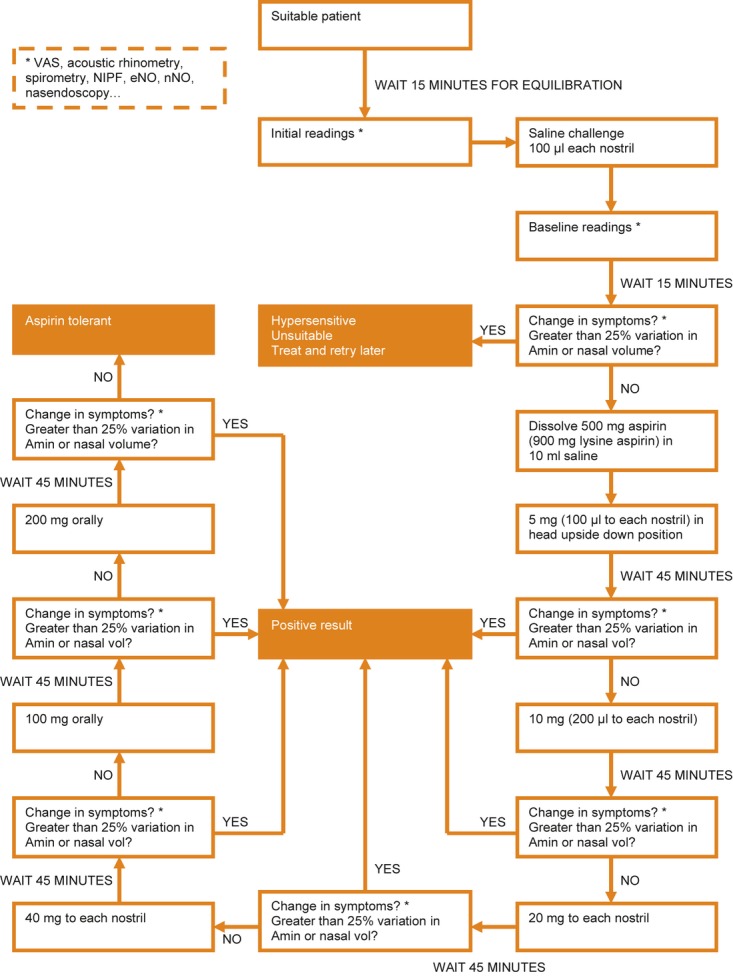
Challenge procedure.

After 15 min, further symptom scores and readings were made and if the change on acoustic rhinometry was greater than 25% decrease in Amin the challenge was abandoned as the nasal hyper-reactivity was too severe to permit accurate diagnosis. The patient was given further more intense anti-inflammatory treatment to reduce hyper-reactivity and re-booked for repeat challenge at least a month later. If the placebo challenge did not cause a significant reduction in the nasal airway then graduated challenge with lysine aspirin was performed using initial doses of 5–10 mg applied as 100 μL in drop form in the head upside down position, one to each nostril, after which the patient remained with head upside down for 1 min. Re-assessment was made at 45 min, and if no significant change had occurred a double dose was administered with further readings after 45 min. If the patient was symptomatic and changes had occurred, but did not reach those needed for a positive challenge then a further set of readings was made after a further interval of 15–30 min. This process was repeated using doses of 20 mg, then 40 mg intranasally according to the algorithm (Fig. 2). The maximal cumulative nasal dose was usually 75–100 mg.

Patients who were negative at the nasal provocation were challenged sublingually with increasing doses of aspirin to reach the cumulative doses of at least 350 mg of aspirin.

## Criteria for a positive challenge response

The criteria were taken from the definitive paper on aspirin challenge 10 and were as follows: Increased symptoms [see Fig. 1 recorded by VAS, plus either 25% or greater decrease in the nasal airway (represented by a reduction of cross-sectional area: Amin or volume 0–12 cm)] as assessed by acoustic rhinometry or a 40% decrease in nasal inspiratory peak flow.

Reproducibility of the nasal challenge was assessed by a second challenge in 83 patients who took a second nasal application of lysine aspirin 24 h later at the dose to which they were previously positive.

## Results

Six non-rhinitic, non-asthmatic control subjects (3 males, age range 35–65) tested with 120 mg nasal lysine aspirin reported initial mild irritation, lasting under 2 min. No other upper or lower airway symptoms or changes occurred.

**Figure 3:**
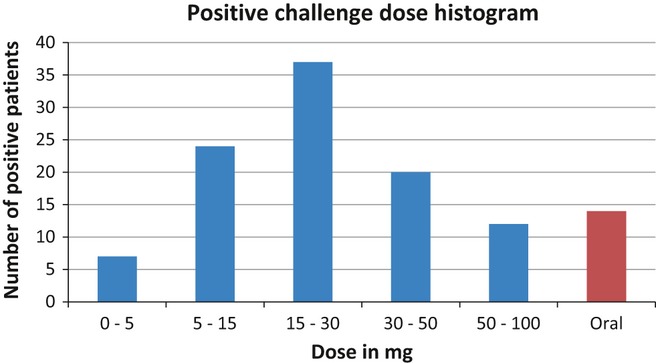
Histogram to show numbers of patients reacting at each intranasal dose of lysine aspirin.

One hundred and fifty patients [76 men and 74 women; mean age 47 ± 13 (SD) years] with chronic rhinosinusitis were recruited from the Rhinology Clinic at the Royal National Throat, Nose and Ear Hospital, London, from 2002 to Spring 2010. Written formal consent was obtained from all the patients. One hundred were positive on nasal challenge at the doses indicated in Fig. 3. There was a 71% correlation between the provoking dose on history and the dose at positive challenge in those with a history of a reaction to aspirin.

Fourteen who were negative on nasal challenge were positive when challenged with higher doses orally – see Fig. 3. Of these, four had to return on a second outpatient visit to complete the challenge, which was re-started at the last previously tolerated dose. Seventeen patients (13%) were ASA tolerant with negative challenge test. Nineteen patients were not challenged as they did not fulfil the inclusion criteria.

All positive patients reacted with nasal symptoms. These were treated with nasal decongestants and topical nasal steroids. Twenty-one patients also developed mild lower respiratory tract symptoms, including mild bronchospam and /or chest tightening,and were treated with beta 2 agonists and inhaled corticosteroid. Two patients with a greater than 20% drop in FEV1 following nasal plus oral challenge were given oral corticosteroid for 3 days in addition.

Seven patients noted symptoms outside the respiratory tract: mild facial itching, urticaria and mild facial angioedema (one patient, see below), with no previous history of these. No one required adrenaline.

One individual had a positive history of aspirin sensitivity, but was negative on challenge. One individual who had not previously suffered this symptom required brief oral corticosteroid (30 mg/day for 3 days) therapy for facial angioedema. In retrospect, it was realized that as he had grade 3 polyps at the start of challenge any response by increase in polyp size was difficult to appreciate and he was given an extra higher dose of lysine aspirin after he had begun to react. Subsequently, we avoided challenging patients with grossly obstructive polyps.

Nasal inspiratory peak flow (NIPF) fell more than 40% in only 15 of 51 (15.7%) patients at the stage where they were positive on acoustic rhinometry, with a 25% decrease in Amin and /or volume 0–12 cm.

Reproducibility of the nasal challenge was good: 81 of 83 patients who took a second nasal application of lysine aspirin 24 h later at the dose to which they were previously positive noted a recurrence of their nasal symptoms and upper airway obstruction.

## Discussion

There is a need for ENT surgeons, allergists and chest physicians to diagnose aspirin sensitivity safely, since there is a need to inform those who need to avoid COX-1 antagonists and to enable the non-aspirin-sensitive to use such medications. Low dose aspirin reduces cardiovascular morbidity and mortality and is also protective against many cancers 18,19. Unfortunately, no *in vitro* method has a sufficiently high sensitivity and specificity 9, which means that aspirin challenges should be undertaken. Various routes are available: oral, bronchial, nasal and intravenous 10–12,20. Oral challenges are expensive and time- consuming, requiring admission of the patient and the initiation of an intravenous infusion since they can be dangerous with severe prolonged asthma exacerbations. The other routes all employ lysine aspirin, the only truly soluble form of aspirin. Bronchial challenges require specialized equipment usually unavailable outside the respiratory department and can also result in severe asthma 11. Intravenous challenges are performed in Japan only and are no faster than topical administration, but carry significant risks 20.

In the ENT setting, there is the specific need and the easy accessibility to target nasal polyps. The administration of intra-nasal lysine aspirin constitutes the most suitable route to challenge these patients.

In addition, nasal challenge offers a relatively simple and safe method for diagnosis since direct application to sensitive tissue occurs, the reaction is easily monitored and, although it produces an increase in nasal symptoms, does not compromise patient safety. The most sensitive individuals are diagnosed using low doses and only the more tolerant need to go on to be challenged orally, thus avoiding hospital bed use. Our small series rules out major adverse events with a rate of 2%, rare events could still occur so close monitoring of these patients by an experienced physician is necessary.

A method of measuring the nasal airway is vital, since subjective assessment is unreliable. If NIPF were the only available method of monitoring the airway then the 36 subjects who were diagnosed as positive on acoustic rhinometry, but whose NIPF had not fallen 40%, as recommended in guidelines 10, would have required further doses of lysine aspirin,with possible worsening of symptoms.

Therefore, we suggest that NIPF is insufficiently sensitive and acoustic rhinometry should be employed when aspirin challenge is an outpatient procedure. Rhinomanometry (GM Instruments, Kilwinning, Scotland) is not likely to be helpful if the nose is more than minimally blocked at the start of challenge since it is difficult to appreciate changes in the airway when the initial flow/pressure curve is flattened 16.

Patient selection is vital with those with a history of very severe immediate-type reactions being excluded as well as those with non-respiratory symptoms in response to aspirin. It is also necessary to have a nasal airway which is capable of being measured and of being reduced by aspirin so hyper-reactors and the nasally completely occluded patients need treatment to remedy these prior to challenge. Asthma control should be established prior to challenge and asthma medications continued.

Our experience over several years shows that nasal challenge is well tolerated and is capable of diagnosing the majority of positive patients (59% in this study) within 3–4 stepwise administrations a process taking around 3–4 h. A further 29% react at higher nasal doses, leaving only a few (12%) remaining genuinely aspirin-sensitive individuals to be picked up by oral challenge. This whole procedure can be fitted into 1 day, provided a start is made in the morning. Three of our four patients needed to return to complete the challenge because of a late start due to long distance travel.

One individual had a negative challenge result, despite a strongly positive history of aspirin-sensitivity. This is not entirely surprising as it is known that the clinical history alone is not fully reliable for the diagnosis of aspirin sensitivity 7 and the patient had taken other drugs at the same time. The importance of co-factors such as underlying infection, exercise or alcohol could have contributed to the original reaction and may have to be taken into consideration. Discounting a mistaken history, this could represent a false negative result due to nasal steroid use or loss of aspirin sensitivity which has been previously reported but is rare. A second challenge after more prolonged abstention from nasal steroids is advisable. Although female gender has been shown to be a risk factor for AERD 2 in our cohort of patients both sexes were equally affected.

The careful use of nasal lysine aspirin challenges in an ENT setting, with co-operation from respiratory physicians, is possible and would improve the detection rate of aspirin-exacerbated respiratory disease, an under-recognized disorder.
